# Horse-Racing Effect and Clinical Trials in Older Persons

**DOI:** 10.3389/fnagi.2014.00175

**Published:** 2014-07-16

**Authors:** Matteo Cesari, Marco Canevelli

**Affiliations:** ^1^Gérontopôle, Centre Hospitalier Universitaire de Toulouse, Toulouse, France; ^2^INSERM UMR1027, Université de Toulouse III Paul Sabatier, Toulouse, France; ^3^Dipartimento di Neurologia e Psichiatria, Sapienza Università di Roma, Rome, Italy

**Keywords:** clinical trials, study design, elderly, frailty, randomization, horse-racing effect, clinical research, methodology

## Introduction

The standard methodologies for the design and conduction of randomized controlled trials (RCTs) are often difficult to be directly applied when older persons compose the target population (Pahor and Cesari, 2012). In fact, specific methodological adaptations are often required to guarantee the feasibility of the trial, ensure the participants’ adherence/compliance to the protocol, and allow the unbiased/proper interpretation of the findings. In this paper, we present the case of a frequently ignored issue potentially affecting the interpretation of results generated by RCTs. In particular, we describe how the health status fluctuations of older persons (especially in the presence of frailty) may bias the randomization procedures. Possible solutions to such phenomenon are also offered.

## Clinical Trials in Older Persons

Randomized controlled trials represent the gold standard for the assessment of efficacy and effectiveness of interventions. Given the socio-demographic trends of our societies, the conduction of trials in older persons is growingly becoming pivotal. However, old age often implies special challenges in the design of intervention studies from methodological, clinical, and social viewpoints (Pahor and Cesari, 2012). In particular, the representativeness of the study sample (and, consequently, the possible future generalization of the study findings) is often affected by the complexity of this population.

The definition of eligibility criteria in the design of RCTs always represents a crucial and delicate step. The choices done at this time can drastically change the results of the study and concur at determining the success of the project. Such step becomes even more difficult when the trial is aimed at exploring age-related conditions. In fact, the (sub)clinical accumulation of deficits occurring with aging leads to pathophysiological modifications potentially mining the “purity” of diseases (Cesari et al., 2013). The age-related reduction of homeostatic mechanisms against entropic forces (or frailty) (Morley et al., 2013) exposes the older organism at multiple and interacting conditions whose clinical manifestations might often be masked or altered (Studenski, 2009). This issue is at the basis of the well-known “evidence based medicine” problem severely affecting the applicability of clinical recommendations and guidelines to elders (Scott and Guyatt, 2010). On one side, we cannot ignore the need of extending as much as possible the study of interventions to the most fastly growing subgroup of our societies (both in absolute as well as relative terms). On the other hand, we also need to acknowledge a certain inadequacy of traditional standards adopted in clinical trials for judging the efficacy of interventions in the most advanced phases of the aging process (Pahor and Cesari, 2012).

## Evolution of Age-Related Conditions

If time plays a major and evident role in the determination of clinical phenotypes, ignoring its importance in the design of the study and the definition of eligibility criteria may severely affect the conduction of the trial and potentially bias its conclusions. This is particularly true for evolving clinical processes characterized by non-linear trajectories, such as the age-related disabling cascade commonly depicted as a sigmoidal decline of physical function or a self-feeding and accelerating vicious cycle (Ferrucci et al., 2002). If the development of a clinical condition does not follow a linear pattern (as frequently happening among older persons) a single-point or mono-dimensional evaluation may not be sufficient to adequately appreciate the health status of the individual. In other words, the history of the condition of interest imposes to take into account the often ignored “horse-racing effect” when designing an *ad hoc* RCT.

## The Horse-Racing Effect

The horse-racing effect has been originally advocated to explain observational studies exploring the increase of clinical parameters (e.g., blood-pressure) with aging (Anonymous, 1981). It postulates the existence of a close correlation between the aging process and the health status as the speed of the horse is related to its position in the race. Nevertheless, a superficial observation of results may lead to arguable conclusions. Thus, for example, the interpretation of results showing that blood-pressure increases with advancing age might meaningfully shift from “the higher they start, the faster they rise” to “the faster they rise, the higher they are” (Peto, 1981). Such contradicting interpretation does not only affect the analysis of results coming from observational studies. The same risk can easily endanger the correct decoding of findings from RCTs.

**Figure 1:**
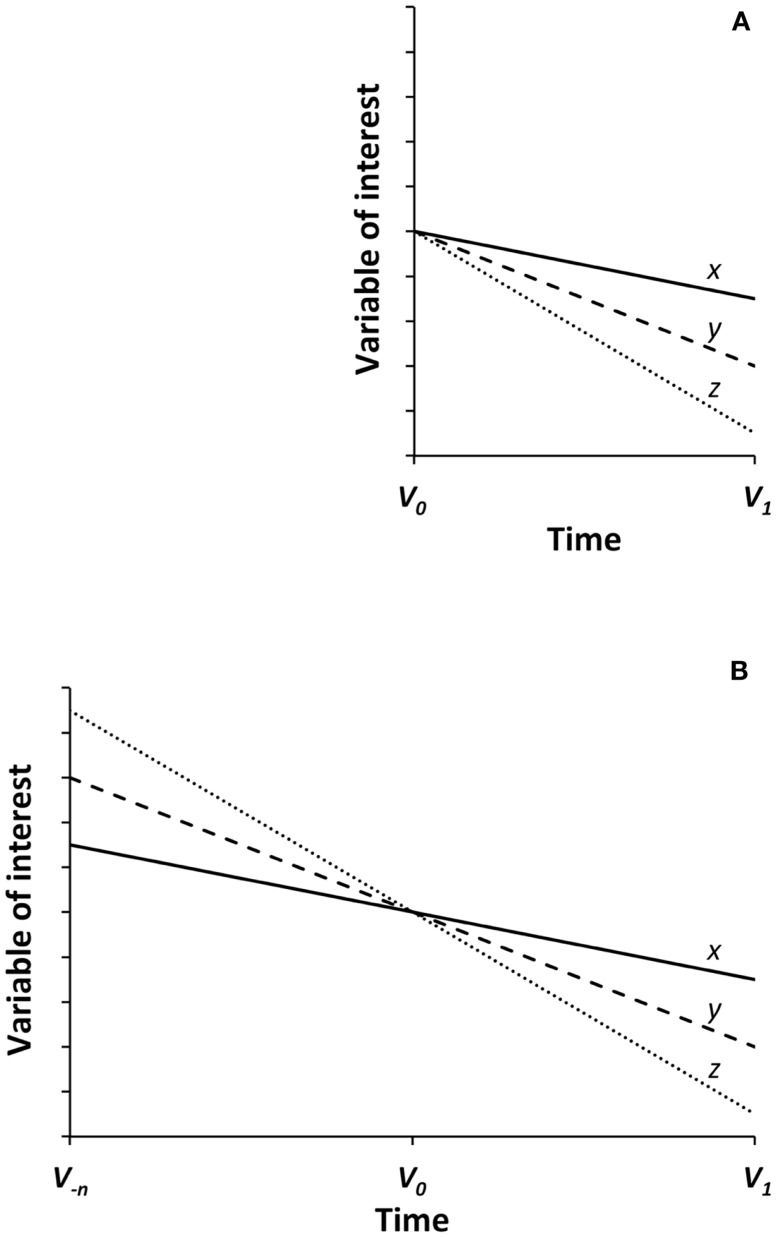
**Graphical description of the “horse-racing effect” in randomized clinical trials**. *V*_0_ and *V*_1_ indicate the assessments conducted at the baseline and follow-up visits of the trial, respectively. *V*_−_*_n_* represents a hypothetical assessment conducted some time before *V*_0_. *x, y*, and *z* describe the trajectories of the variable of interest for the three randomization groups of the trial. Although the three groups (*x, y*, and *z*) seem to be correctly randomized and starting at a similar level at the baseline visit **(A)**, they are indeed characterized by pre-existing differences in their trajectories **(B)**. Such differences significantly affect the interpretation of trial results.

In Figure 1, a graphical description of the relevance of the horse-racing effect in RCTs is provided. Figure 1A depicts the characteristic and schematic overview of results from a RCT. At the time 0 visit (*V*_0_), participants are randomized according to a major clinical characteristic rendering the intervention groups as much homogeneous as possible in relationship with the study outcome. Let’s say, for example, a Mini Mental State Examination (MMSE) score ≥24 to explore the risk of incident dementia. After two or more interventions are conducted for a certain period of time, we can judge the effects of the different trial arms by comparing the difference of the key variable of interest between *V*_1_ (end of the trial visit) and *V*_0_ (baseline visit). Such approach is legitimate and frequently adopted, but presents a high risk of biased results due to: (1) the limited observation of the phenomenon of interest, and (2) the fluctuating value of the variable of interest. In fact, the randomization procedure according to a specific and single criterion may not adequately take into account what happened before the observation began in the three groups. It is like looking at a picture shot at the photo-finish during a horse race: we get the idea about the winner, but our conclusion is justified only because we are sure that horses indeed began running together and from the same stating-point at the gunshot. Translating such concept into the reality of RCTs, it becomes evident that the observation period (i.e., trial follow-up) is infinitesimally shorter compared to the race already covered before the baseline visit (i.e., participant’s life-course), especially if the subject is an older person. In other words, Figure 1A becomes arguable and misleading if what happened before the baseline visit is not adequately considered. In fact, the positive effect of an intervention (*x*) compared to the others (*y* and *z*) might be simply be explained by the less steep decline that participants in that group were already experiencing before the randomization phase. If the observation could be left-extended to any previous time-point *V*_−_*_n_* (Figure 1B), the interpretation of the *x, y*, and *z* trajectories will lead to completely different conclusions (i.e., lack of relevant effects). After all, since between two points only one line can be drawn, the identification of a third point (e.g., *V*_−_*_n_*) in the appreciation of results will allow to determine possible knots of flection and provide a more accurate evaluation of results.

Just to give an example, a cluster-randomized trial was conducted to explore the effectiveness of a specific care plan in patients with mild-to-moderate Alzheimer’s disease (Nourhashemi et al., 2010). The main eligibility criteria of the trial were the possible or probable diagnosis of Alzheimer’s disease, and MMSE score between 12 and 26. As also mentioned by the Authors among the study limitations, the selection of participants (largely relying on the MMSE results for judging the cognitive status) might have been biased the entry criteria, and potentially affected the homogeneity of the sample.

## How to Take into Account the Horse-Racing Effect in Clinical Trials

In order to allow the correct interpretation of RCTs (especially for age-related conditions), the clinical, behavioral, biological, and social experiences occurred before the study start cannot be overlooked. The definition of the entry criteria based on a multiple-point observation (for example, a test administered twice at the distance of some time to guarantee the stability of the condition) might represent a solid method for limiting the “horse-racing effect.” As an alternative, it might be chosen to combine a double evaluation of potential participants using the variable of interest (e.g., MMSE score) in conjunction with a more global measure of disease severity (e.g., Clinical Dementia Rating), with this latter somehow serving as surrogate of exposure time to the risk condition. It should not even be underestimated the role that specific measures estimating the aging status of the individual may play in this context. For example, the Frailty Index proposed by Rockwood and colleagues (measuring the age-related deficit accumulation of the individual) (Rockwood et al., 2005) or the usual gait speed (intended as an additional vital sign) (Cesari, 2011) may support the results of the measured phenomenon by providing additional information about the global health status of the participant.

It might be thought that the randomization of participants in the different arms of the clinical trial might be sufficient to take into account the horse-racing effect. In fact, it is likely that the random allocation of participants to the study interventions might also equally distribute their characteristics and underlying conditions, and consequently the “abnormal” trajectories. This is not completely true. The randomization procedures may reduce the risk of an unequal distribution of participants’ characteristics, but cannot be considered foolproof, especially for those variables that are not object of specific stratification. Moreover, the randomization does not act on the clear and optimal definition of the sample population, but is simply aimed at guaranteeing the fair comparison across groups. Thus, if the horse-racing effect is not adequately addressed in the eligibility criteria of the trial participants, the resulting groups might be similar (thanks to the randomization), but still potentially include completely different conditions (e.g., MMSE = 25 may indicate a persons with early signs of neurodegenerative disorder as well as a person with poor education).

Last but not least, it is important to raise awareness about the inadequacy of the standard methodology adopted in traditional RCTs (targeting adults) when this is applied to studies recruiting older participants (Pahor and Cesari, 2012). The complexity of the older person (especially in the presence of geriatric conditions) indeed requires special adaptations capable to taking into account his/her extreme vulnerability to stressors. In particular, researchers should understand the necessity of shifting from a disease-oriented approach (typical of RCTs in adults) to a holistic and function-oriented one in order to design informative and robust RCTs in older persons (Studenski, 2009).

## Conclusion

The “horse-racing effect” described in the context of observational studies of aging represents a major source of confounding in RCTs, too. Researchers should become more aware about the risks of conducting one-point and mono-dimensional assessments at the recruitment phase of intervention studies targeting age-related conditions.

## Conflict of Interest Statement

The authors declare that the research was conducted in the absence of any commercial or financial relationships that could be construed as a potential conflict of interest.
